# Analysis of factors associated with abnormal thyroid function among medical staff in radiotherapy departments

**DOI:** 10.3389/fpubh.2023.1225879

**Published:** 2023-08-17

**Authors:** Ziwei Ren, Yanwei Ren, Xinghua Bai, Puyu Shang, Guang Li

**Affiliations:** ^1^Department of Radiotherapy, The First Hospital of China Medical University, Shenyang, Liaoning, China; ^2^Department of Paediatrics, The First Hospital of China Medical University, Shenyang, Liaoning, China

**Keywords:** abnormal thyroid function, radiotherapy, depression, stress, occupation exposure

## Abstract

**Objective:**

To understand abnormal thyroid function and its associated factors among medical staff in radiotherapy departments.

**Methods:**

Data related to medical staff in radiotherapy departments who met the inclusion and exclusion criteria were analyzed from September to December 2022 in Shenyang, Liaoning Province, including basic personal and lifestyle habits, work information, and scores on the Depression Self-Assessment Scale, Perception Stress Scale, and the Generalized Anxiety Scale. Data analysis was performed using the χ^2^ test and binary logistic regression.

**Results:**

Among 484 medical staff in the radiotherapy department, 147 (30.4%) had abnormal thyroid function. Binary logistic regression analysis showed that age, years of work, smoking, occupational exposure, smoking, late-night snacking habits, depression, and stress perception were factors associated with abnormal thyroid function among medical staff in radiotherapy departments; on the other hand, physical exercise was a protective factor.

**Conclusion:**

The positive rate of thyroid dysfunction among medical personnel in the radiology department is relatively high. It is necessary to strengthen health education and awareness among relevant practitioners and improve the coverage of occupational disease prevention and control education.

## 1. Introduction

Abnormal thyroid function is closely associated with prolonged occupational exposure, which is more common among medical staff in radiation therapy units (1). It is an endocrine disorder caused by insufficient synthesis secretion/biological effect or excessive release of thyroid hormones due to low or high thyroid action (2). Abnormal functional status increases the risk of anxiety, anger, obesity, diabetes, hypertension, and thyroid cancer among doctors and nurses (3), which adversely affects patient care, such as inappropriate protective measures leading to exposure to low-dose radiation, emotional instability of doctors and nurses leading to tension between doctors and patients, and long-term suboptimal health of doctors and nurses affecting the treatment plan (4, 5). Therefore, it is important to explore the abnormal thyroid function of medical staff in radiotherapy departments and its associated factors to promote harmony between doctors and patients and maintain the physical and mental health of medical and nursing staff.

Research studies have found that the incidence of thyroid function abnormalities among medical staff is between 4 and 30% (6, 7), showing an increasing trend year by year. Factors influencing thyroid function abnormalities include demographic factors (age, gender, education, etc.), work factors (level of protection, type of work, length of service, etc.), disease factors (coronary heart disease, diabetes, dyslipidemia, etc.), and lifestyle habits (smoking, drinking, dietary preferences, etc.) (8–11). However, studies on the association between factors such as psychological regulation, behavioral patterns, physical exercise, and thyroid function abnormalities among medical staff in radiotherapy departments are less common.

Currently, abnormal thyroid function is becoming an important public health and occupational health problem threatening the health of radiologists. Although medical staff are aware of the hazards of low-dose radiation and thyroid disease, they need to analyze potential risk factors and put interventions and preventive measures in place. This study investigates the association between abnormal thyroid function and its associated factors among medical staff in radiotherapy departments, especially exposure to lifestyle, psychological regulation, and behavioral patterns, to provide a scientific basis for preventing and controlling occupational disease hazards.

## 2. Methods

### 2.1. Study design

This study adopted a cross-sectional design, using the random number table method to randomly select 10 hospitals from 37 public tertiary A hospitals in Shenyang, Liaoning Province, from September to December 2022. Approximately 50 medical staff from the radiation therapy departments of the 10 hospitals were randomly sampled, and all individuals who met the standards were included in the target population.

### 2.2. Participant

The sample size of the study was calculated using the formula: *N* = 400 × *Q*/*P* (*Q* = 1-*P* and *P* was the incidence rate). Due to the lack of a unified incidence rate of abnormal thyroid hormone levels, after consulting Chinese domestic literature (12), the detection rate of abnormal levels of thyroid hormones among medical staff was set at 46.6%, and *N* = 458 people were calculated. Considering the possibility of invalid and missing questionnaires in the investigation process, 500 questionnaires were issued, of which 484 were recovered, with a recovery rate of 96.8%. All questionnaires were valid, so the target population of this study was set at 484 people. Inclusion criteria: (1) Aged ≥18 years; (2) Abnormal function as determined by two or more internists by thyroid color Doppler ultrasound and serum thyroid hormone tests [triiodothyronine (T3), thyroxine (T4), and thyrotropin (TSH)]; (3) Voluntary participation in this study and no participation in similar studies within 6 months; and (4) No mental disorders caused by cerebrovascular diseases, physical infections, or other physical diseases. Exclusion criteria: (1) Family history of thyroid disease, history or other autoimmune diseases; (2) Have a history of pituitary or hypothalamic diseases; (3) Have a history of severe parathyroid disease; (4) Chronic consumptive diseases (diabetes, abnormal liver function, abnormal kidney function, cardiac insufficiency, chronic infectious diseases, etc.) and malignant tumors; (5) Recent history of acute and severe infections; (6) Take or use drugs that affect thyroid hormones, such as phenytoin sodium, Carbamazepine, Amiodarone, Lithium carbonate, and iodine-containing contrast agent, and (7) Pregnant women. The study was approved by the hospital's ethical review committee, and all participants gave informed consent to the study content.

### 2.3. Instrumentation

#### 2.3.1. General information survey

The questionnaire covered basic personal details, living habits, and work information. The basic personal information and living habits included gender, age, education, family history of thyroid disease, alcohol consumption, smoking, frequency of physical exercise, and whether there was a night snacking habit. The basic work information included years of work, title, type of occupation, whether the night shift was on rotation, and whether occupational exposure occurred.

#### 2.3.2. Abnormal thyroid function

The five indicators of thyroid function included free triiodothyronine (CFT3), free thyroxine (CFT4), thyroid stimulating hormone (TSH), thyroglobulin antibody (TGAb), and thyroid peroxidase antibody (TPOAb). Approximately 3–5 ml of fasting peripheral blood was collected from the study participants and placed in a non-anticoagulant tube. It was let to stand at room temperature for 30 m and centrifuged at 3,500 rpm for 10 m, with a centrifugal force of 1,095^*^g. Approximately 500 uI of serum was collected and placed in an EP tube. Simultaneously, a backup was created and immediately frozen in a freezer at −20°C for testing. In this study, five indicators of thyroid function in the serum of the participants were detected by Roche E411 automatic electrochemiluminescence immune analyzer and its detection reagent. All experimental reagents were calibrated using a standard calibration solution, and those that passed the calibration were used on the machine. The products were tested daily for quality control before the experiments to ensure that each experiment was within the quality control range. The reference ranges of the blood biochemical indexes were: TSH 0.27–4.2 mIU/L, FT4 12–22 pmol/L, FT3 3.1–6.8 pmol/L, TPOAb 3–34 mIU/L, and TGAb 0–115 mIU/L. Two internists jointly determined whether the thyroid function was abnormal based on the blood biochemical index results according to the Chinese Guidelines for the Diagnosis and Treatment of Thyroid disease.

#### 2.3.3. The Generalized Anxiety Disorder (GAD-7)

The scale consists of seven symptoms and one symptom-related degree of difficulty item that assessed the severity and functional impact of the seven anxiety symptoms over the past 2 weeks (13). Each symptom was rated on a 4-point Likert scale from 0 (not at all) to 3 (almost every day). The degree of difficulty degree item had four options (no difficulty at all, somewhat difficult, very difficult, and extremely difficult) but was not included in the score. The total score of the scale was 21, with 0–4 indicating no anxiety, 5–9 mild anxiety, 10–14 moderate anxiety, and ≥15 suggesting severe anxiety. This questionnaire has been widely used in the Chinese population and has good reliability and validity (14, 15). The Cronbach's alpha for this scale in this study was 0.851 (GFI = 0.982, RMSEA = 0.027, CFI = 0.995, NFI = 0.982).

#### 2.3.4. Depression Self-Assessment Scale (PHQ-9)

The scale was developed based on the nine symptoms of DSM-IV depressive disorder and was widely used in scientific research and clinical practice because of its brevity and ease of use (16). The total score of the PHQ-9 ranges from 0 to 27, with scores of 5, 10, and 20 being the cut-off values for the degree of mild, moderate, and severe depression, respectively. The scale has good sensitivity and specificity in the screening and diagnosis of depression (17). This questionnaire has been widely used in the Chinese population and has good reliability and validity (14, 18). The Cronbach's alpha for this scale in this study was 0.910 (GFI = 0.964, RMSEA = 0.032, CFI = 0.988, NFI = 0.964).

#### 2.3.5. The perceived stress scale

The perceived stress scale (PSS) was developed by Cohen et al. (19) in 1983 and is a more widely used tool for assessing perceived stress. This study used the Chinese version of the perceived stress scale (CPSS) based on PSS-14 (20). It is simple and easy to use and is mainly used to measure the level of stress in an individual's life. It consists of two dimensions: a sense of loss of control and a sense of tension, with 14 items, also known as the PSS-14. Each item is rated on a 5-point Likert scale, ranging from 0 to 4 on a scale of “strongly disagree” to “strongly agree.” Items 4, 5, 6, 7, 9, 10, and 13 were scored inversely, with a total score ranging between 0 and 56; higher scores indicated greater perceived stress. This questionnaire has been widely used in the Chinese population and has good reliability and validity (21, 22). The Cronbach's alpha for this scale in this study was 0.952 (GFI = 0.968, RMSEA = 0.020, CFI = 0.995, NFI = 0.968).

### 2.4. Statistical analysis

All collected data were analyzed using SPSS 26.0 software. The count variables were expressed as percentages, and comparisons between groups were made using the χ^2^ test. The measured variables were expressed as mean ± standard deviation, and a *t*-test was used for comparison between groups. Univariate and multivariate logistic regression were used to determine the factors influencing abnormal thyroid function.

## 3. Results

### 3.1. Basic information of the participants

A total of 500 questionnaires were issued, and 484 valid questionnaires were returned, including 147 with abnormal thyroid function, with a detection rate of 30.4%. Hyperthyroidism, hypothyroidism, subclinical hyperthyroidism, and subclinical hypothyroidism were detected in 15, 28, 37, and 67 cases, with detection rates of 10.2%, 19.0%, 25.2%, and 45.6%, respectively. The age range of medical staff in the radiotherapy department was 21–37 years old, with an average of 29.0 ± 8.6 years. There were 283 (58.5%) female and 201 (41.5%) male staff. Approximately 289 participants mainly had irregular eating habits, accounting for 51.9%. In addition, 224 (46.3%) and 226 (46.7%) medical doctors had smoking and drinking habits, respectively. Approximately 216 (44.6%) participants never exercised, 112 (23.1%) occasionally exercised, and 156 (32.3%) always exercised. Their occupational types were predominantly physicians 168 (34.7%), in addition to 99 (20.5%) nurses, 122 (25.2%) physiatrists, 46 (9.5%) technicians, and 49 (10.1%) other staff. Approximately 287 (59.3%) of the staff had a history of occupational exposure. Most of the participants had worked for 5 years or less, and those with intermediate and below job titles were predominant. There were 319 (65.9%) medical staff involved in night shifts and 165 (34.1%) staff without night shifts. The details are shown in Table 1.

**Table 1:** Basic information of the participants.

**Variables**	**Category**	**Total**	**Non-abnormal thyroid function (*n* = 337)**	**Abnormal thyroid function (*n* = 147)**	**χ^2^**	***p*-value**
Age					40.698	< 0.001
	18–25	143	129	14		
	26–40	271	166	105		
	≥41	70	42	28		
Gender					1.215	0.270
	Male	275	197	78		
	Female	209	140	69		
Education level					0.864	0.834
	Junior	116	80	36		
	Undergraduate	149	108	41		
	Master	102	76	36		
	Doctor	107	73	34		
Working years					24.880	< 0.001
	< 3	112	95	17		
	3–5	143	104	39		
	6–10	102	73	39		
	≥11	117	65	52		
Occupation					11.601	0.021
	Physician	168	107	61		
	Nurse	99	75	24		
	Physicist	122	80	42		
	Technician	46	33	13		
	Other	49	42	7		
Job title					4.140	0.126
	Primary	353	254	99		
	Intermediate	70	42	28		
	Senior	61	41	20		
Night shift					7.478	0.006
	No	165	128	37		
	Yes	319	209	110		
Occupational exposure					17.567	< 0.001
	No	197	158	39		
	Yes	287	179	108		
Smoking					11.313	0.001
	No	260	198	62		
	Yes	224	139	85		
Drinking alcohol					0.845	0.358
	No	258	175	83		
	Yes	226	162	64		
Night snack habits					16.877	< 0.001
	No	233	183	50		
	Yes	251	154	97		
Family history of thyroid disease					1.354	0.245
	No	289	207	82		
	Yes	195	130	65		
Physical exercise					28.718	< 0.001
	Never	216	124	92		
	Occasional	112	85	27		
	Always	156	128	28		

### 3.2. Anxiety, depression, and stress perceptions of medical staff in the radiotherapy department

Reviewing the depression, anxiety, and stress assessments in both groups, the results showed that the PHQ-9, GAD-7, and PSS scores were significantly higher in the abnormal thyroid functioning group than in the non-abnormal group, and the differences were statistically significant. The details are shown in Table 2.

**Table 2:** Anxiety, depression, and stress perception of radiotherapy medical staff.

**Variables**	**GAD-7**	**PHQ-9**	**PSS**
Non-abnormal thyroid function	9.13 ± 4.70	12.93 ± 5.42	31.44 ± 8.64
Abnormal thyroid function	11.71 ± 5.03	17.24 ± 5.64	39.50 ± 13.56
*t*	−5.437	−7.591	−6.646
*p*-value	< 0.001	< 0.001	< 0.001

### 3.3. Univariate logistic regression analysis of factors associated with abnormal thyroid function among medical staff in radiotherapy departments

The results of univariate logistic regression analysis showed that age, years of work, type of occupation, job title, night shift, occupational exposure, late-night snacking habit, smoking, family history, physical exercise status, GAD-7, PHQ-9, and PSS were associated with abnormal thyroid function among medical staff in the radiotherapy department. The details are shown in Table 3.

**Table 3:** Univariate logistic regression analysis of the factors associated with thyroid dysfunction in the radiotherapy department.

**Variables**		***B***	***SE***	***Wald***	***OR***	***95% CI***	***p*-value**
Age		0.045	0.011	17.916	1.046	1.025, 1.068	< 0.001
**Gender**							
	Male	Reference					
	Female	0.219	0.199	1.213	1.245	0.843, 1.838	0.271
**Education level**							
	Junior	Reference		0.863			0.834
	Undergraduate	−0.17	0.272	0.391	0.844	0.495, 1.437	0.532
	Master	0.051	0.285	0.032	1.053	0.602, 1.840	0.857
	Doctor	0.034	0.289	0.014	1.035	0.588, 1.823	0.905
**Working years**							
	< 3	Reference		23.452			< 0.001
	3–5	0.74	0.323	5.233	2.096	1.112, 3.950	0.022
	6–10	1.094	0.33	11.007	2.985	1.565, 5.697	0.001
	≥11	1.498	0.322	21.57	4.471	2.376, 8.411	< 0.001
**Occupation**							
	Physician	Reference		11.026			0.026
	Nurse	−0.577	0.284	4.130	0.561	0.322, 0.980	0.042
	Physicist	−0.082	0.249	0.109	0.921	0.565, 1.501	0.741
	Technician	−0.370	0.365	1.027	0.691	0.338, 1.412	0.311
	Other	−1.230	0.439	7.861	0.292	0.124, 0.691	0.005
**Job title**							
	Primary	Reference		4.090			0.129
	Intermediate	0.537	0.271	3.916	1.710	1.005, 2.911	0.048
	Senior	0.224	0.297	0.569	1.252	0.699, 2.242	0.451
**Night shift**							
	No	Reference					
	Yes	0.599	0.221	7.372	1.821	1.181, 2.806	0.007
**Occupational exposure**							
	No	Reference					
	Yes	0.894	0.216	17.063	2.444	1.600, 3.735	< 0.001
**Smoking**							
	No	Reference					
	Yes	0.669	0.200	11.161	1.953	1.319, 2.892	0.001
**Drinking alcohol**							
	No	Reference					
	Yes	−0.183	0.199	0.844	0.833	0.564, 1.230	0.358
**Night snack habits**							
	No	Reference					
	Yes	0.835	0.206	16.504	2.305	1.541, 3.449	< 0.001
**Family history of thyroid disease**							
	No	Reference					
	Yes	0.233	0.200	1.352	1.262	0.852, 1.869	0.245
**Physical exercise**							
	Never	Reference		27.568			< 0.001
	Occasional	−0.848	0.260	10.624	0.428	0.257, 0.713	0.001
	Always	−1.221	0.250	23.881	0.295	0.181, 0.481	< 0.001
GAD		0.110	0.021	26.706	1.116	1.070, 1.163	< 0.001
PHQ		0.143	0.020	50.609	1.153	1.109, 1.199	< 0.001
PSS		0.082	0.012	48.450	1.085	1.060, 1.100	< 0.001

### 3.4. Multivariate logistic regression analysis of factors associated with abnormal thyroid function among medical staff in radiotherapy departments

A multivariate logistic regression analysis was performed with abnormal thyroid function (0 = no, 1 = yes) as the dependent variable and factors with *p* < 0.1 in the univariate logistic regression analysis as independent variables. The results showed that age, years of work, occupational exposure, smoking, late-night snacking habits, depression, and stress perception were associated factors for abnormal thyroid function among medical staff in the radiotherapy department: physical exercise, on the other hand, was a protective factor for abnormal thyroid function. For details, see Table 4.

**Table 4:** Multivariate logistic regression analysis of factors associated with thyroid function abnormalities in the radiotherapy department.

**Variables**		***B***	***SE***	***Wald***	***OR***	***95% CI***	***p*-value**
Age		0.304	0.034	78.356	1.356	1.267, 1.450	< 0.001
**Working years**							
	< 3	Reference		12.075			0.007
	3–5	−0.427	0.494	0.749	0.652	0.248, 1.717	0.387
	6–10	0.553	0.509	1.176	1.738	0.640, 4.716	0.278
	≥11	1.023	0.486	4.431	2.781	1.073, 7.207	0.035
**Occupation**							
	Physician	Reference		4.830			0.305
	Nurse	−0.648	0.460	1.986	0.523	0.212, 1.288	0.159
	Physicist	−0.069	0.416	0.028	0.933	0.413, 2.108	0.868
	Technician	−0.351	0.619	0.322	0.704	0.209, 2.368	0.571
	Other	−1.250	0.682	3.361	0.287	0.075, 1.090	0.067
**Job title**							
	Primary	Reference		2.090			0.352
	Intermediate	0.407	0.443	0.842	1.502	0.630, 3.580	0.359
	Senior	−0.462	0.536	0.743	0.630	0.220, 1.802	0.389
**Night shift**							
	No	Reference					
	Yes	−0.140	0.374	0.140	0.869	0.418, 1.808	0.708
**Occupational exposure**							
	No	Reference					
	Yes	0.904	0.344	6.888	2.469	1.257, 4.850	0.009
**Smoking**							
	No	Reference					
	Yes	0.968	0.329	8.672	2.632	1.382, 5.012	0.003
**Night snack habits**							
	No	Reference					
	Yes	1.310	0.344	14.524	3.706	1.889, 7.268	< 0.001
**Physical exercise**							
	Never	Reference		15.839			< 0.001
	Occasional	−0.610	0.430	2.013	0.543	0.234, 1.262	0.156
	Always	−1.696	0.426	15.834	0.183	0.080, 0.423	< 0.001
GAD		−0.067	0.045	2.176	0.935	0.856, 1.022	0.140
PHQ		0.116	0.043	7.293	1.123	1.032, 1.222	0.007
PSS		0.248	0.033	54.653	1.281	1.199, 1.368	< 0.001

## 4. Discussion

The thyroid gland is the largest endocrine gland in adults, which mainly regulates the growth and development of the human body, and affects emotions and the metabolism of sugar, fat, and protein. The prevalence of thyroid disease is increasing year by year (23). This study found that medical staff are more likely to suffer from thyroid disease than ordinary people (24). It also found that the detection rate of abnormal thyroid function was 30.4% based on the results of thyroid function testing and a questionnaire survey among 484 medical personnel in the radiotherapy department in Liaoning Province. The detection rate was significantly higher than the 6.6% surveyed in Europe and the United States (2) and higher than the 25% in the community-based older population aged 65 years and older in the United States (25). The positive rate of thyroid function abnormalities in this study was lower than the 39.4% reported by Zhang et al. (26) for medical radiologists in Nanjing but higher than the 12.3% reported by Santos et al. (27) for permanent residents aged 20 years and older in South America. The differences in study results may be due to differences in economic development, social environment, thyroid function measurement tools, and the participants included in different countries and regions. The literature search revealed that there are few studies on the factors associated with abnormal thyroid function in medical staff in radiotherapy departments, and most studies focus on physical and chemical indicators or functional changes in the thyroid gland but less on the influence of social, psychological, and behavioral factors on abnormal thyroid function (28–31).

The results of the multivariate analysis showed that age and working hours were associated factors for abnormal thyroid function in medical staff in radiation therapy units—the older the medical staff in radiation therapy units and the longer the working years, the higher the positive rate of abnormal thyroid function among them, which is generally consistent with previous studies (29, 32). The analysis suggests that with the accumulation of low-dose radiation exposure, thyroid peroxidase expression decreases, leading to inhibition of thyroid hormone synthesis (33), which, in turn, accelerates the incidence of thyroid dysfunction. The present study's results showed that the risk of thyroid function abnormalities among medical staff in radiotherapy departments with previous occupational exposure was 2.4 times higher than those without occupational exposure.

The thyroid gland is sensitive to ionizing radiation, and long-term exposure can lead to dysfunction and teratogenic effects (34). Radiation can cause vascular damage, parenchymal cell damage, and autoimmune cell response in the thyroid gland (35). Ron and Brenner (36) found that ionizing radiation increases the risk of goiter and thyroid nodules, and the incidence of benign thyroid nodules and follicular thyroid adenoma had a significant dose-effect relationship with ionizing radiation. The results of a cross-sectional survey based on the community population in Pomerania showed that occupational ionizing radiation exposure is related to the risk of autoimmune thyroid disease (37). The study found that medical staff did not take thyroid protection seriously during their prolonged professional practice and that staff who did not wear thyroid protection were frequently exposed to ionizing radiation, thus making the thyroid more vulnerable to radiation injury (31).

The results of this study showed that the risk of thyroid function abnormalities was higher in radiotherapy medical staff who smoked than in nonsmokers and that their serum TSH levels were significantly higher than in nonsmokers (38). This is probably because the alkaloids and nicotine in tobacco cause the body to be in a chronic inflammatory state and induce cellular mediators (produced by immune cells) that can accelerate the deterioration of thyroid function (39). Late-night snacking habits are also considered to be one of the risk factors affecting thyroid function. Studies have shown that regular and reasonably arranged intake of meat, staple foods, vegetables, and fruits are beneficial for preventing thyroid function disorders (40), which is consistent with the findings of this paper. A Mediterranean characteristic diet, low in animal foods and high in plant foods, is more protective for thyroid function than a high animal foods diet (41) and may be related to the balanced gain in nutrient intake from a good diet. Similarly, stress perception is a risk factor for abnormal thyroid function in radiotherapy medical staff, probably because acute or chronic stressful stress induces an imbalance in the body's immune system (42, 43), leading to an overproduction of glucocorticoids and catecholamines through activation of the thalamus-pituitary-adrenal axis, which, in turn, leads to the development of abnormal thyroid function. Depression has also been found to be a risk factor for abnormal thyroid function in medical staff in radiotherapy departments. The stimulation of adverse psychosomatic factors such as depression can cause disorders of the neuroendocrine system, affecting the abnormal function of the HPA axis, impeding thyroid hormone secretion, and affecting the normal functioning of the thyroid gland (28).

Medical staff who exercise daily are less likely to have abnormal thyroid function than those who exercise less than twice a week, which may be related to continuous and regular exercise reducing resting T4, T3, and thyroid-stimulating hormone levels. Further, it has been noted that exercise can alter the levels of zinc in the body, which is considered an important substance involved in synthesizing and processing thyroid hormones (44, 45).

## 5. Implications and limitations

This study is an important addition to the epidemiology of thyroid disorders. With the rising incidence of cancer, the issue of abnormal thyroid function among medical staff in radiotherapy units deserves attention and prevention. Age, years of work, smoking, occupational exposure, late-night snacking habits, depression, and perceived stress were found to be risk factors for abnormal thyroid function among radiotherapy medical staff, with physical activity as a protective factor. The study still has the following limitations. First, the sample size was limited, and all were from the same study center, subject to selection bias. Second, cross-sectional studies, which do not provide good evidence of causality, are necessary to conduct cohort studies to assess the effect of each factor more clearly on abnormal thyroid function.

## 6. Conclusions

In conclusion, the positive rate of thyroid dysfunction among medical personnel in the radiology department is relatively high. It is necessary to strengthen health education and awareness among relevant practitioners and improve the coverage of occupational disease prevention and control education. Hospitals should conduct regular training to enhance the radiation protection awareness of medical personnel, focusing on protecting the thyroid area and improving protective conditions. Medical staff should cultivate good living habits and maintain their mental health.

## Data availability statement

The original contributions presented in the study are included in the article/Supplementary material, further inquiries can be directed to the corresponding authors.

## Ethics statement

The studies involving humans were approved by the Ethics Committee of The First Hospital of China Medical University. The studies were conducted in accordance with the local legislation and institutional requirements. The participants provided their written informed consent to participate in this study. Written informed consent was obtained from the individual(s) for the publication of any potentially identifiable images or data included in this article.

## Author contributions

ZR and PS: full access to all the data in the study, take data analysis, and supervision. YR: conceptualization, methodology, and writing—original draft. XB: software and validation. GL: writing—reviewing and editing. All authors approved the submitted version.
